# Intralesional injection of tuberculin purified protein derivative (PPD) versus measles, mumps, and rubella (MMR) vaccine in treatment of molluscum contagiosum: a comparative study

**DOI:** 10.1038/s41598-023-49182-2

**Published:** 2024-01-02

**Authors:** Mohamed S. Zaky, Rabie B. Atallah, Aya M. Saad Mohyeldeen, Mohamed L. Elsaie

**Affiliations:** 1 Department of Dermatology, Venereology and Andrology, Damietta Faculty of Medicine, Al-Azhar University, Damietta, Egypt; 2 Department of Dermatology, Venereology and Andrology, Medical Research and Clinical Studies Institute, National Research Centre, Giza, Egypt

**Keywords:** Diseases, Medical research

## Abstract

Molluscum contagiosum (MC) is a skin and mucous membrane infection caused by the molluscum virus (MCV). To evaluate safety and efficacy of intralesional injection of tuberculin purified protein derivative (PPD) antigen injection versus MMR (mumps, measles, rubella) antigen for the treatment of molluscum contagiosum (MC). A total of thirty clinically confirmed patients of molluscum were recruited for this trial. Patients who were divided into three groups (A, B and C). Each group consisted of (30) patients. Group (A) subjects received intralesional MMR injections, group (B) subjects received intralesional PPD injection and group (C) received intralesional saline injection. The results of the present study revealed complete clearance of the injected lesions in 12 patients (80%), partial response in 3 patients (20%) of group (A). In group (B), complete clearance of the treated warts was observed in 11 patients (73.3%) and partial response in 4 (26.7%) of patients. In group (C), the majority of patients 8 (53.3%) demonstrated no response while 7 (46.7%) patients showed only partial clearance. We established a good safety and efficacy profile for tuberculin PPD and MMR antigens in treatment of molluscum contagiosum.

## Introduction

Molluscum contagiosum (MC) is a skin and mucous membrane infection caused by the molluscum virus (MCV)^1^. MCV is a DNA poxvirus that causes a cutaneous infection in children and immune suppressed individuals. Resolution of lesions is possible over time; yet it may take months and years for lesions to clear up spontaneously in immune competent individuals. Four types of MCV (1–4) are implicated in lesion appearance of which 1 is the commonest and 2 is frequent in adults^2^.

MC is highly contagious and spread by direct contact with the infected skin or mucous membrane which can be sexually or non-sexually transmitted or even via autoincoculation. Among children fomite spread through infected sponges or towels in swimming pools is widely described^3^.

No available consensus or FDA approved treatment exists for MC and majority of treatments remain to be destructive in origin. A plethora of treatments including cautery, cryotherapy, lasers, topical application of cantharidin, trichloroacetic acid, imiquimod as well as curettage had been used with variable results and a number of side effects^3^.

The innate immune response in MC infection has not been extensively studied. Lately, intralesional immunotherapy has been used extensively in warts^4^. Considering the role of cell-mediated immunity in the pathogenesis of MC, intralesional immunotherapy could be promising in clearing epidermal infected keratinocytes. Intralesional immunotherapy is a safe treatment option with minimal adverse effects. The procedure was tolerated well in all ages^4^.

The advisory committee on immunization practices in Egypt recommends 2 compulsory doses of MMR vaccine for all children. The first dose of MMR 0.5 ml vaccine should be administered to all children beginning at or after age 12 months, and the second dose of 0.5 ml routinely at 12 months of age. As for BCG vaccination a compulsory dose of 0.05 ml is given by intradermal injection at 0–40 days of age^5^.

To our knowledge no studies compared MMR and PPD for treating MC. Therefore we aimed to further evaluate the efficacy, safety and tolerability of both antigens compared to each other and to a control group in clearing MC lesions.

## Material and methods

A total of thirty clinically confirmed patients of molluscum were recruited for this trial from April 2022 to November 2022. Participants were recruited randomly from the outpatient clinic of the Dermatology and Venerology department et al.-Azhar University Hospital in New Damietta, Egypt. The study was approved by the Damietta Faculty of Medicine Al-Azhar University's Research Ethics Committee (00012367-21-02-002). All participants or their guardians gave their informed consent to participate in the trial^6^.

Patients of MC who were immune competent, with history of BCG vaccination, above 3 years of age and under no concurrent systemic or topical treatment of MC within the past 6 weeks was included. Those with history of asthma, febrile illness, immunosuppressive conditions or allergic skin disorders were not included. Moreover pregnant or lactating females were excluded from the study.

Demographic details including age and sex were recorded. Careful medical history and clinical examination as well as baseline characteristics of molluscum lesions, including number, size, and site involved, and duration were recorded at the start of the study and each follow-up visit. Appropriate digital photographs were taken before the start, at each visit and after completion of treatment.

The patients were divided randomly using sealed envelope method into three groups. In group (A) patients, 0.1 ml of the MMR antigen was first injected into the forearm and only positive reactors (showing ≥ 5 mm induration at the injection site within 48–72 h of testing) were included in the study. Included subjects were then injected with 0.1 ml (10 IU) of MMR vaccine (Trimovax Merieux-Aventis, 0.5 ml) in their biggest lesion using an insulin syringe held parallel to the skin surface, with the bevel facing upward. Group (B) included 15 patients who were injected intralesionally with 0.1 ml (10 IU) of PPD tuberculin (VACSERA®, Egypt 2 ml vial) in the largest lesion. Group (C) included 15 patients who were injected intralesionally with 0.1 ml of normal saline (Otsuka®, Egypt) in the largest lesion. Treatments were carried out every three weeks and for 3 treatments or until full clearance whichever was achieved first. Following each treatment patients were instructed to remain at the clinic for 30 min for possible signs of immediate hypersensitivity. Patients were followed up for two months from the last treatment session for any sign of recurrence^6^.

Complete response was considered if lesions had disappeared completely while partial response was considered if regression in lesion size or of more than 50% and no response was considered if lesions persisted as is during the full treatment period (9 weeks). Side effects during the course of treatment as pain, itching erythema, edema, induration, ulceration and general systemic action as flu-like symptoms were recorded.

Data were fed to the computer and analyzed using IBM SPSS Corp. Released 2013. IBM SPSS for Windows® version 22.0 Armonk, NY: IBM Corp. Qualitative data were described using number and percent. Quantitative data were described using median (minimum and maximum) and mean, standard deviation for parametric data after testing normality using Kolmogrov-Smirnov test. Significance of the obtained results was judged at the (0.05) level.

### Sample size calculation

To estimate the sample size, we used Open Epi program Version 3 and according to: MC response rate (outcome) with Tuberculin Purified Protein Derivative (PPD) 85%, and to investigate the preference of intralesional immunotherapy injection with Measles, Mumps, and Rubella (MMR) Vaccine over Tuberculin Purified Protein Derivative (PPD) and placebo. Assuming alpha error is 5%, 95% confidence level and the study power is 80%. Sample size was established to be at least 7 patients for each study group. To compensate for possible 25% drop off or failure to follow up 15 subjects were selected for each group.

### Study approval and consent to participate

This study protocol was reviewed and approved by ethics committee on human research by Al Azhar faculty of medicine (IRB 00012367-21-02-002). All methods were performed in accordance with the relevant guidelines and regulations. Written informed consents were received from participants upon explanation of the study. Consent for publication was obtained from the participants for publishing the images in the manuscript.

## Results

Group A (MMR) included 7 males (46.7%) and 8 females (53.3%) and their ages ranged from 4 to 28 years with median of 16 years. Group B (PPD) included 9 males (60%) and 6 females (40%) with a median age of 6 years while group C (Saline) included 7 males (46.7%) and 8 females (53.5%) with ages ranging from 4 to 20 years and a median of 8 years. All three groups were comparable to one another and no drop outs were reported. No difference was recorded between the three groups in terms of participants age (p1 = 0.370, p2 = 0.607, p3 = 0.700; respectively) and gender (p1 = 0.464, p2 = 1, p3 = 0.464 respectively). Tables 1 and 2

**Table 1:** Socio-demographic characteristics of the studied groups.

	MMR group N = 15	PPD group N = 15	Saline group N = 15	Test of significance	Within group significance
Age/years	16 (4–28)	6 (4–18)	8 (4–20)	KW = 0.413	P1 = 0.370
Median (range)				P = 0.664	P2 = 0.607
					P3 = 0.700
Sex n (%)					P1 = 0.464
Male	7 (46.7)	9 (60)	7 (46.7)	χ^2^ = 0.711	P2 = 1.0
Female	8 (53.3)	6 (40)	8 (53.3)	P = 0.701	P3 = 0.464

KW, Kruskal Wallis test; χ^2^, Chi-Square test; P1, difference between MMR & PPD group; P2, difference between MMR & Saline group; P3, difference between PPD & Saline group, within group significance was done by Mann Whitney U test.

**Table 2:** Lesion characteristics distribution among the studied groups.

	MMR group N = 15	PPD group N = 15	Saline group N = 15	Test of significance	Within group significance
Duration of disease (months)	4 (2–6)	3 (2–6)	4 (2–6)	KW	P1 = 0.226
				*P* = 0.446	P2 = 0.8066
					P3 = 0.367
Number of lesions	11 (6–16)	10 (6–17)	8 (6–12)	KW	P1 = 0.492
				*P* = 0.051	P2 = 0.06
					P3 = 0.081
Site					
Trunk	4 (26.7)	4 (26.7)	5 (33.3)	χ^2^ = 11.35	
Leg	0	2 (13.3)	2 (13.3)	*P* = 0.499	P1 = 0.306
Face & neck	9 (60)	8 (53.3)	5 (33.3)		P2 = 0.375
Elbow	0	0	1 (6.7)		P3 = 0.785
Arm	1 (6.7)	1 (6.7)	2 (13.3)		
Abdomen	1 (6.7)	0	0		

KW, Kruskal Wallis test; χ^2^, Chi-Square test; P1, difference between MMR & PPD group; P2, difference between MMR & Saline group; P3, difference between PPD & Saline group, within group significance was done by Mann Whitney U test.

In all three groups duration of MC lesions insignificantly ranged from 2 to 6 months. The majority of lesions in all groups were distributed on the face and neck (60%, 53.3%, and 33.3% respectively) as well as on the trunk (33.4%, 26.7%, and 33.3% respectively). Fewer lesions were distributed on the extremities. No significant differences were reported in all groups regarding site (p1 = 0.306, p2 = 0.375, p3 = 0.785; respectively) or number of the lesions (p1 = 0.492, p2 = 0.065, p3 = 0.081; respectively).

**Figure 1:**
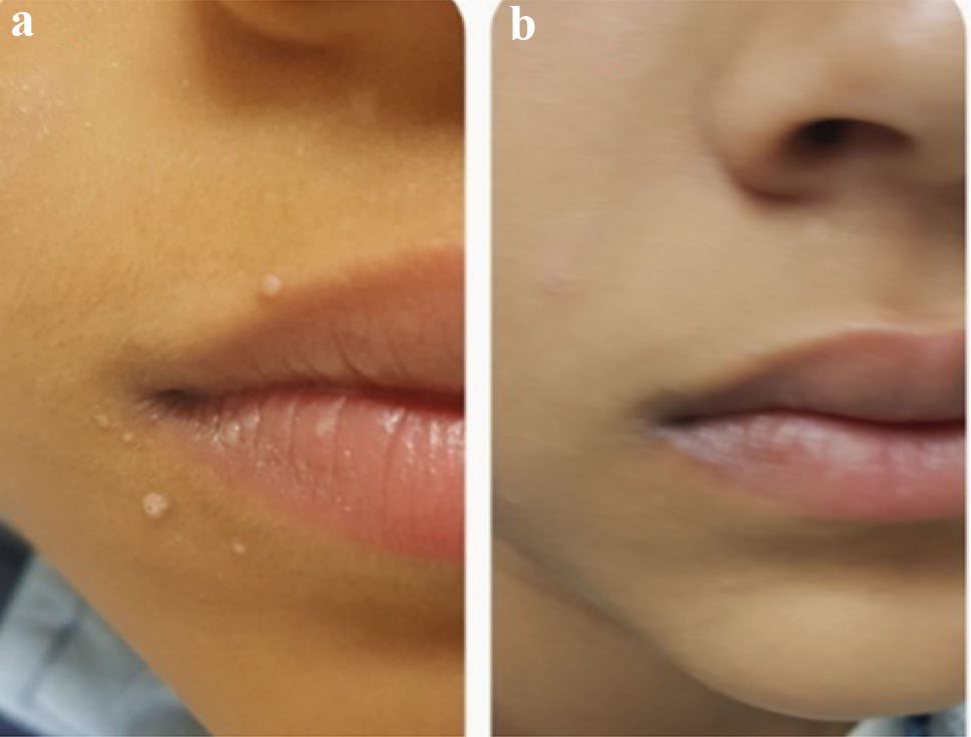
A 14 years old male with facial MC (**a**) before and (**b**) after treatment with three MMR treatments showing disappearance of the lesions.

**Figure 2:**
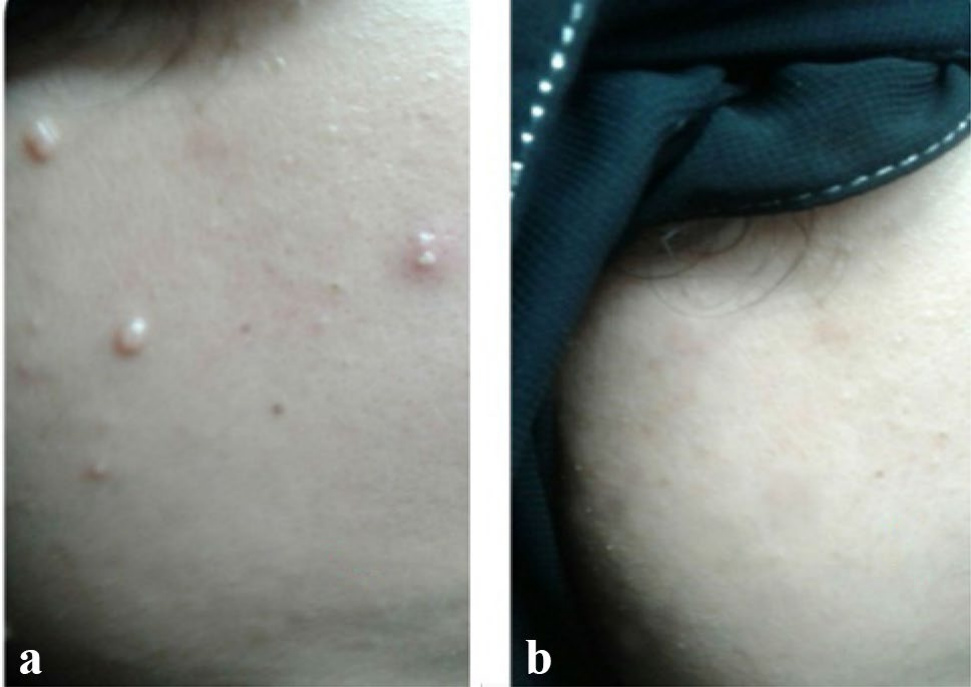
A 20 years old female with facial MC (**a**) before and (**b**) after treatment with three PPD treatments showing disappearance of the lesions.

The results of the present study revealed complete clearance of the injected lesions in 12 patients (80%), partial response in 3 patients (20%) of group (A). In group (B), complete clearance of the treated warts was observed in 11 patients (73.3%) and partial response in 4 (26.7%) of patients. In group (C), the majority of patients 8 (53.3%) demonstrated no response while 7 (46.7%) patients showed only partial clearance. Comparison between groups A and B to control group C demonstrated significant rates of wart clearance (*p* < 0.001). Notably, no significant difference between the MMR and PPD-treated groups was demonstrated (*p* = 0.666). By the end of the 2 months follow up, recurrence of lesions was reported insignificantly in 3 cases of group A (MMR) and 4 cases of group B (PPD) (*p* = 0.666). In the MMR and PPD-treated groups, a significant full clearance response was achieved in patients with less number of lesions (*p* = 0.009, *p* = 0.004 respectively). Similarly significant recurrence rates were demonstrated in subjects with more number of lesions in both MMR and PPD treated groups (*p* = 0.009, *p* = 0.004 respectively). Tables 3, 4, 5 and 6; Figs. 1 and 2.

**Table 3:** Clinical response and recurrence among the studied groups.

	MMR group N = 15	PPD group N = 15	Saline group N = 15	Test of significance	Within group significance
Clinical response					
No	0	0	8 (53.3)	χ^2MC^ = 29.42	P1 = 0.666
Partial	3 (20)	4 (26.7)	7 (46.7)	*P* < 0.001*	P2 < 0.001*
Complete	12 (80)	11 (73.3)	0		P3 < 0.001*
Recurrence	3 (20)	4 (26.7)	15 (100)	χ^2MC^ = 23.66	P1 = 0.666
				*P* < 0.001*	P2 < 0.001*
					P3 < 0.001*

χ^2^, Chi-Square test; MC, Monte Carlo test; P1, difference between MMR & PPD group; P2, difference between MMR & Saline group; P3, difference between PPD & Saline group.

* : statistically signifcant.

**Table 4:** Complications distribution among the studied groups.

	MMR group N = 15	PPD group N = 15	Saline group N = 15	Test of significance	Within group significance
Erythema	15 (100)	15 (100)	15 (100)		
Pain	15 (100)	15 (100)	15 (100)		
Fever	15 (100)	15 (100)	0	χ^2MC^ = 45.0	P1 = 1.0
				*P* < 0.001*	P2 < 0.001*
					P3 < 0.001*

χ^2^, Chi-Square test; MC, Monte Carlo test; P1, difference between MMR & PPD group; P2, difference between MMR & Saline group; P3, difference between PPD & Saline group.

* : statistically signifcant.

**Table 5:** Predictors of clinical response among the MMR group.

	Clinical response for MMR group		Test of significance
	Partial response N = 3	Complete response N = 12	
Age/years	5 (4–9)	7 (4–28)	Z = 0.876
Median (range)			*P* = 0.381
Sex			
Male	2 (66.7)	5 (41.7)	FET
Female	1 (33.3)	7 (58.3)	*P* = 0.569
Disease duration	4 (2–6)	4 (2–6)	Z = 0.074
Median (range)			*P* = 0.941
Number of lesions	15 (15–16)	10 (6–14)	Z = 2.61
Median (range)			*P* = 0.009*
Site			
Trunk	1 (33.3)	3 (25)	MC
Face & neck	1 (33.3)	8 (66.7)	*P* = 0.277
Arm	0	1 (8.3)	
Abdomen	1 (33.3)	0	

Z, Mann Whitney U test; FET, Fisher exact test; MC, Monte Carlo test.

* : statistically signifcant.

**Table 6:** Predictors of clinical response among the PPD group.

	Clinical response for PPD group		Test of significance
	Partial response N = 4	Complete response N = 11	
Age/years	8 (4–18)	6 (5–12)	Z = 0.067
Median (range)			*P* = 0.947
Sex			
Male	1 (25)	8 (72.7)	FET
Female	3 (75)	3 (27.3)	*P* = 0.235
Disease duration	4 (3–5)	2 (2–6)	Z = 1.086
Median (range)			*P* = 0.277
Number of lesions	14 (12–17)	9 (6–11)	Z = 2.89
Median (range)			*P* = 0.004*
Site			
Trunk	3 (75)	1 (9.1)	MC
Leg	1 (25)	1 (9.1)	*P* = 0.072
Face & neck	0	8 (72.7)	
Arm	0	1 (9.1)	

Z, Mann Whitney U test; FET, Fisher exact test; MC, Monte Carlo test.

* : statistically signifcant.

### Adverse effects

Initial tolerable and transient pain was the most frequent complaint that recovered completely with using of nonsteroidal anti-inflammatory drugs within 24 h. Similarly injection site erythema was encountered in all three groups. Moderate to high fever (38–40 °C) occurred in all subjects of groups A and B mostly after the initial injection and were controllable. Healing process were insignificant among group A and B subjects and no other reports of ulceration, indurations or scarring were reported.

## Discussion

Taking into consideration the innate immune response in the development of MC lesions, a number of earlier studied assessed different immnune therapies for treating MC^2,7–14^. Table 7.

**Table 7:** Studies of previous immunotherapeutics in molluscum contagiosum.

Author	Treatment type	No. of patients	Average age in years (range)	Therapy dosage	Number of treatments	Complete response (%)	Partial response (%)	Recurrence rate (%)	Most common complication
Maronn et al.^7^	Candida	25	5.04 (2–15)	0.3 mL	3.07	56	28	Unknown	Erythema
Enns and Evans ^8^	Candida	29	5.14 (2–17)	0.3 mL	2.5 (max 6)	55.2	37.9	0	None
Gamil et al.^9^	Candida	16	6.93 (2–18)	0.3 mL	4–5	50	25	0	Localized edema
Bakke and Stein^10^	Candida	1	1.58	Unknown	3	100	n/a	0	None
Thomas et al.^2^	Candida	1	70	0.3 mL	4	100	n/a	0	None
Na et al.^11^	MMR	2	2 (1–4)	0.3 mL	3	100	n/a	0	None
Chahun et al.^12^	MMR	22	19 (6–50)	0.1–0.3 mL	3	81.8	18.2	0	None
Khattab and Nasr ^13^	PPD	20	13 (3–40)	0.1 mL	4 (max 6)	85	15	25	Edema and erythema
Sonthalia et al.^14^	Vit D	2	13	0.2 mL	2	100	n/a	0	None

Intralesional MMR vaccine for recalcitrant MC was only reported in two previous studies. Na and his colleagues reported complete resolution of MC by injecting 0.3 ml of MMR into the largest lesion of 2 pediatric patients with no signs of recurrence or adverse events^11^. In their recent study Chauhan et al.^12^ reported treating 22MC patients (10 males and 12 females) with 0.1 ml of intralesional MMR for three sessions. At the end of 12 weeks, 18 patients (81.8%) had complete clearance of lesions, with 4 patients (18.18%) having a partial response of more than 50%. No side effects were reported apart from hyper pigmentation in only one case^12^.

The exact mechanism to which MMR targets MC virus remains to be fully elucidated. Intralesional MMR is believed to alter the immune system to achieve a targeted, specific delayed-type hypersensitivity immune response to both the vaccine proteins as well as MC virus leading to sustained control of viral proliferation^15^. It had been established that virus infected keratinocytes in the epidermis of the skin result in release of tumor necrosis factor β and interferon α via their increased expression of toll like receptors. MMR injections alleviate Th1 response and destroy viral infected cells^1^.

Only one trial found tuberculin PPD to be as significantly equivalent, effective and tolerable when compared to topical cantharidin for treating MC. Moreover 0.2 ml of vitamin D3 was intralesionally injected into the largest molluscum lesion of 2 patients in 2 separate sessions separated by 4 weeks and complete clearance of the lesions was secured in both cases with no reportable side effects^13^.

Efficacy of intralesional immunotherapy in MC with candida antigen has been reported in a few studies. One study on a pediatric population complaining of MC demonstrated a 56% complete clearance and 28% partial clearance after 3 treatments of 0.3 ml candida antigen delivered 4 weeks apart^7^. Candida antigen had been one of the early investigated antigens for treating warts and with clearance rates approaching 75%^16,17^.

The antigen stimulates the immune system to respond to the virus at the injection site as well as distally. While the exact mechanism of action has not been fully elucidated, the injected Candida antigen is thought to induce a T helper 1 (Th1) response with production of cytokines leading to activated T cell mediated elimination of the virally infected cells both locally and at distant sites^18^.

Sonthalia et al. reported the use of intralesional vitamin D immunotherapy in two immunosuppressed patients with persistent widespread MC lesions. In both patients, two of the larger lesions were injected at their base with 0.2 ml of vitamin D3 using a 23 G needle and the treatment was repeated after one month. In both patients complete clearance was observed after the 2nd injection and with no recurrence for a 6 months follow up^13^. The exact mechanism of intralesional vitamin D3 in MC may be similar to that postulated in warts such as regulating epidermal cell proliferation, differentiation, cytokine modulation, and toll-like receptor-mediated genetic induction of antimicrobial peptides^18,19^.

Limitations to the study were the relatively smaller sample size of included subjects and the relatively short follow up period. To our knowledge this is the first study comparing MMR and PPD for treating MC. In conclusion we demonstrated the efficacy, safety, tolerability and cost effectiveness of both tuberculin PPD and MMR antigens in clearing resistant MC with minimal side effects. To achieve an optimal response with an ideal immunotherapeutic agent and ideal dose, further comparative large scale multicenter studies are warranted to verify the study findings.

## Data Availability

The data that support the findings of this study are available from the corresponding author upon reasonable request.
